# To gain insights into heat adaptations among women during the antenatal period in India -The Heat in Pregnancy Project (HiP-I) India- Qualitative Study Protocol

**DOI:** 10.12688/wellcomeopenres.25507.2

**Published:** 2026-04-24

**Authors:** Chris Mary Kurian, Karthika Kumar, Diana Thomas, Yogesh Jain, Sudhakar Reddy Bulla, Gabriela De Jesus Cipriano Flores, Surya Surendran, Prakriti Dayal, Sreevatsan Raghavan, Mudita Gosain, Ramachandran Thiruvengadam, Arya Thonikund Sathishkumar, Stalin Prabhakaran, Vidhya Venugopal, Nitya Wadhwa, Shinjini Bhatnagar, Devaki Nambiar, Jane E Hirst, Praveen D, Heat in Pregnancy India Consortium

**Affiliations:** 1 The George Institute for Global Health India, New Delhi, Delhi, India; 2 The George Institute for Global Health UK, Oxford, England, UK; 3 Translational Health Science and Technology Institute, Faridabad, Haryana, India; 4 Pondicherry Institute of Medical Sciences, Puducherry, Puducherry, India; 5 Sri Ramachandra Institute of Higher Education and Research (Deemed to be University), Chennai, Tamil Nadu, India

**Keywords:** Extreme heat, climate change, pregnancy, climate adaptation, India, qualitative research, gender, socio-ecological framework

## Abstract

**Background:**

Climate change is increasing the frequency and severity of heatwaves across South Asia, with disproportionate impacts on vulnerable populations. Pregnant and lactating women face heightened health risks due to physiological changes, gendered social responsibilities, and constrained access to adaptive resources. Despite this, women’s lived experiences and adaptation practices during pregnancy remain understudied and largely absent from institutional climate responses. This knowledge gap hampers the development of effective, gender-sensitive adaptation policies.

**Methods:**

This qualitative study, embedded within the Heat in Pregnancy (HiP)-India project, will be conducted in three climatically vulnerable sites: Gurugram (Haryana), Bilaspur (Chhattisgarh), and Puducherry. Guided by a socio-ecological framework, we will conduct in-depth interviews with pregnant and lactating women, focus group discussions with caregivers, and key informant interviews with health workers and local stakeholders, alongside non-participant observations. Interviews will take place during and shortly after the heat season to capture both real-time and reflective experiences. Using purposive sampling, we will recruit 20–25 women per site, along with caregivers and stakeholders, primarily from the HiP-India cohort who have consented to follow-up. Data will be thematically analysed using NVivo, with reporting guided by COREQ standards.

**Anticipated Results:**

The study will generate contextualised insights into women’s heat-adaptation practices during pregnancy and lactation across diverse agro-climatic zones, document traditional and emerging coping strategies, and identify structural, social, and institutional barriers shaping adaptation capacity.

**Conclusion:**

By centering women’s lived experiences, this research will inform the design of culturally appropriate, gender-responsive heat adaptation interventions suitable for low-resource settings. Findings will support both practical community-based solutions and evidence-based advocacy for more inclusive climate adaptation policies at local and national levels.

## Plain Language Summary

**Heat Resilience Among Pregnant and Breastfeeding Women in India**

As climate change intensifies across India, extreme heat that threatens pregnant women’s health and their babies’ development. Despite their heightened vulnerability, their specific needs remain largely overlooked in both climate adaptation planning and healthcare systems.

Recognising this critical gap, researchers from the Heat in Pregnancy (HiP) consortium have launched the HiP-India project to better understand how women navigate these challenges. The study will take place during the summers of 2025–2026 across three diverse locations — Gurugram (Haryana), Bilaspur (Chhattisgarh), and Puducherry — representing different environmental and cultural contexts within India.

The research team will talk directly with pregnant and breastfeeding women to understand their daily experiences and how they adjust to heat. They will also speak with family members, community health workers, and local leaders to gain a better understanding of the support available and the challenges women face in staying safe during hot weather.

The findings will show how heat affects maternal health and what kinds of support women need. The main goal is to inform better policies and programs that meet the needs of women during pregnancy and early motherhood by identifying practical, affordable, and locally relevant solutions that build resilience and improve health outcomes.

## Introduction

Rising global temperatures, primarily driven by anthropogenic activities, are both a cause and a consequence of climate change. ^1^ One of the most significant manifestations of this phenomenon is the increasing frequency, intensity, and duration of heat waves. The Intergovernmental Panel on Climate Change (IPCC) 2023 report highlights South Asia as a critical climate hotspot, projecting a sharp rise in the frequency and severity of heatwaves across the region in the coming decades. ^2^

Heatwaves, typically defined as prolonged periods of exceptionally high temperatures, are often accompanied by elevated humidity and vary by region. ^3^ The Indian Meteorological Department (IMD) defines a heatwave as temperatures exceeding 40 °C in the plains or 30 °C in hilly areas, or when temperatures are significantly higher than the normal seasonal average for a given area. ^4^^,^^5^ While the devastating 2010 Ahmedabad heat wave, which caused over 1,300 deaths, catalysed the development of India’s first Heat Action Plan, the burden of heat-related mortality continues to rise. ^6^^,^^7^ In 2024, India endured 77 days of heat wave conditions, nearly double the previous year, resulting in 210 heat-related deaths. ^8^

The health consequences of extreme heat extend beyond immediate heat-related illnesses like dehydration and heat stroke to include exacerbation of cardiovascular and renal diseases. ^9^ Growing evidence links extreme heat exposure during pregnancy to adverse outcomes, including preterm birth, low birth weight, and stillbirth, though the mechanisms remain incompletely understood. ^10^^–^^12^ These impacts are not distributed equally across populations, with exposure and health outcomes shaped by intersecting socioeconomic, occupational, and geographic factors that compound existing health disparities. ^13^

Understanding these complex interactions requires a recognition that heat adaptation occurs across multiple interconnected levels of influence. ^14^^,^^15^ At the individual level, pregnancy and lactation increase physiological vulnerability through altered thermoregulation, higher metabolic demands, and greater fluid requirements. ^16^^,^^17^ A person’s understanding of these risks and the actions they take, such as staying hydrated, resting, and seeking cooler environments, directly influence their ability to protect themselves from extreme heat and reduce potential health impacts. These individual risks, however, are shaped by broader factors, including family support and resource allocation, community-level infrastructure and social systems, and institutional policies that either enable or constrain protective behaviours. ^15^ The intersection of these multi-level influences creates a complex landscape of vulnerability that pregnant and lactating women must navigate during extreme heat events. ^15^

This complexity may be acute for women in low- and middle-income countries like India, especially those from marginalised communities where many women engage in manual labour and unpaid domestic work, often in poorly ventilated environments with minimal access to cooling infrastructure. ^18^ These occupational realities, combined with constrained access to resources and sociocultural barriers, amplify heat exposure while limiting adaptive capacity. Structural barriers to healthcare and environmental services further intensify these challenges, creating a cascade of vulnerability that operates across individual, household, and community levels. ^19^ For pregnant and lactating women, these gendered vulnerabilities intersect with additional physiological stress, making them particularly susceptible to heat-related health impacts. However, in a country like India, the ability to protect against these risks depends not only on individual physiology but also on family support systems, community resources, and institutional responses that either facilitate or constrain protective behaviours. ^19^^,^^20^

Despite these complex, interconnected vulnerabilities, women’s experiences and coping strategies during pregnancy and lactation remain overlooked in climate adaptation planning and maternal health programmes. ^21^ In India, although women are recognised as a vulnerable group, proposed interventions often lack specificity, especially for maternal health. ^21^^–^^23^ These policies frequently lack targeted maternal health protocols, fail to ensure access to cooling technologies, and overlook traditional adaptation practices. ^24^ This gap reflects broader limitations in how heat vulnerability is understood and addressed, particularly the tendency to focus on individual risk factors rather than the multi-level systems that shape adaptive capacity.

The global literature on heat and health has focused on high-income contexts, with limited attention to the lived experiences of women in LMICs. ^11^^,^^25^ Quantitative approaches, while valuable, often overlook the complex social, cultural, and behavioural dimensions of heat adaptation that operate across individual, household, community, and institutional levels. A qualitative systematic review of pregnancy-related heat experiences underscores this gap directly. Drawing on just 16 studies and almost none from South Asia; finds that women’s adaptation strategies are largely reactive, low-cost, and driven by socio-economic necessity rather than health system support. ^26^ How these strategies emerge, are sustained, and interact across levels of the social ecosystem remains poorly understood, yet this understanding is precisely what is needed to design interventions that are effective and contextually grounded.

Heat in Pregnancy – India (HiP-India) is a collaborative, interdisciplinary research initiative aimed at understanding how extreme heat contributes to adverse pregnancy outcomes. ^27^ Led by researchers from institutions in India and the UK, the project integrates cutting-edge approaches from climate science, medical imaging, and laboratory diagnostics to examine the effects of heat exposure on maternal health, placental and fetal function, and lactation.

In this paper, we describe the rationale and methods of a study nested within the HiP-India Program. ^28^ In this study, we aim to explore the adaptation strategies employed by pregnant and lactating women in response to extreme heat in India, and to understand how environmental, sociocultural, and economic factors interact to shape maternal vulnerability and resilience.

Our objectives aligns with the levels of the WHO Socioecological framework, and aim to capture both the experiences of pregnant and lactating women and the actions they are able to take in response to extreme heat. ^14^ Specifically, at the (i) individual level: To document the lived experiences of pregnant and lactating women during the summer months at HiP-India study sites, including their understanding of heat-related risks and the strategies they employ to protect themselves; (ii) family level: to examine the effect of heat on families with pregnant and lactating women and identify opportunities for adaptation at the family level; (iii) community/organizational level: to identify how communities and health institutions adapt to high temperatures and determine adaptation opportunities for pregnant and lactating women; and (iv) policy level: to analyse what heat adaptation plans and climate change policies exist at local and state level to protect pregnant and lactating women from extreme heat.

## Methodology

**Figure 1.**
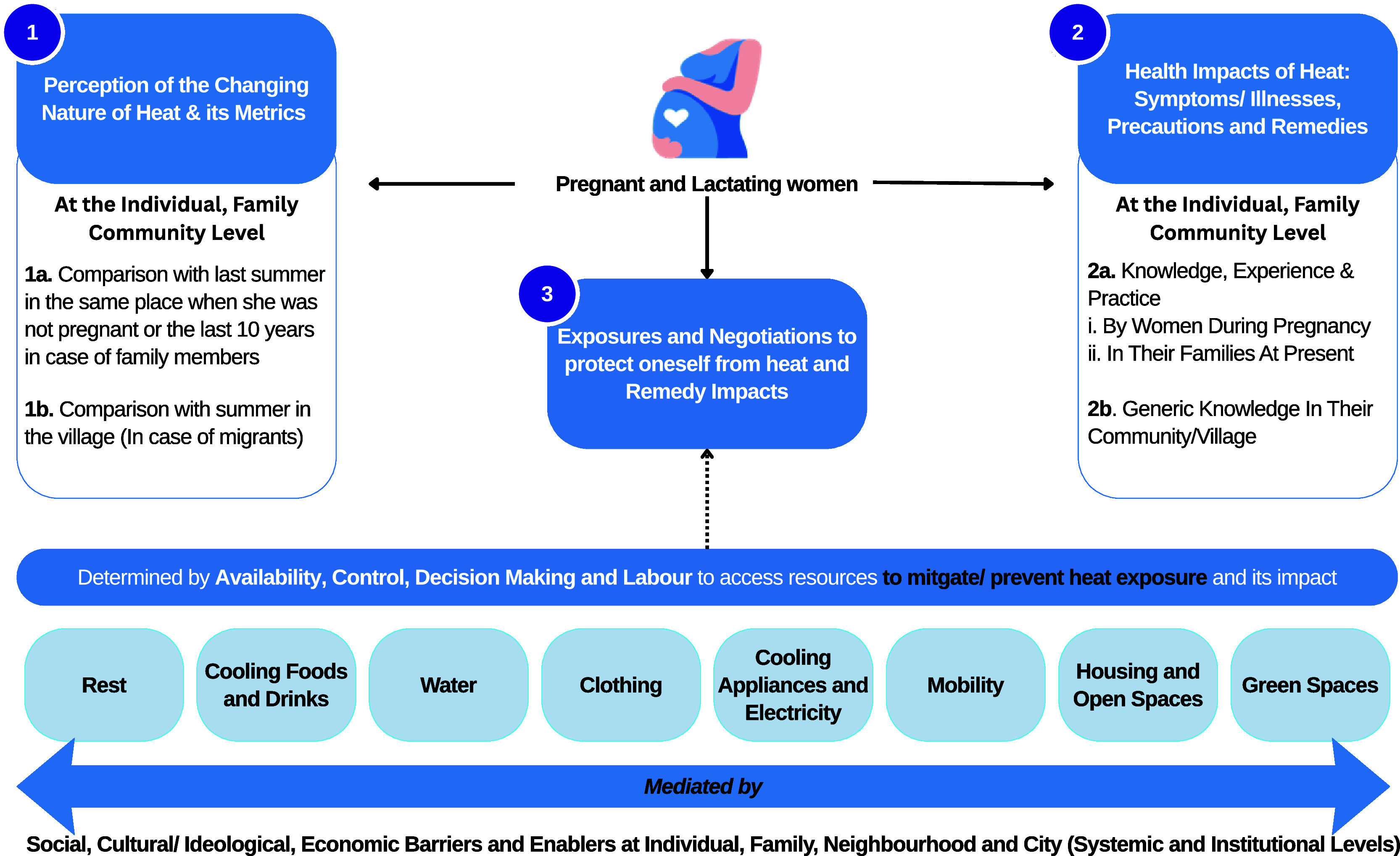
Study Conceptualisation.

This study employs an exploratory qualitative research design to examine adaptation strategies among pregnant and lactating women experiencing extreme heat events in India. Nested within the HiP-India prospective cohort study, the research utilises a feminist approach to investigate women’s lived experiences and identify culturally appropriate, locally relevant coping mechanisms. Adopting the socio-ecological framework, it examines heat-related vulnerability and resilience across multiple levels of influence: individual, family, community, institutional, and policy domains ( Figure 1). By centering women’s everyday experiences in climate-affected environments, this research seeks to inform the development of inclusive, context-sensitive maternal health interventions for climate adaptation.

## Setting and participants

This qualitative study will be conducted across three sites Gurugram (Haryana), Bilaspur (Chhattisgarh), and Puducherry within the HiP-India prospective cohort. These sites represent diverse urban and peri-urban settings across distinct agro-climatic zones in India, each characterised by high heat exposure but differing in socio-environmental conditions, population characteristics, and health system contexts. This diversity enables both within-site and cross-site comparisons to better understand how context shapes women’s vulnerability and adaptive responses to heat. Prior to the data collection, a round table held with local experts in each site will help map the local context within which the women are embedded, to facilitate the analysis of the data.

Neighbourhoods and communities included in the qualitative study will be identified based on geographic clustering within the consenting cohort participants. This approach facilitates efficient data collection by focusing on one or two proximate neighbourhoods or villages per site, while also ensuring that the sample remains embedded within the broader cohort.

Participants will include currently pregnant women purposively selected from the cohort of 200 women enrolled at each site in the HiP-India study. Participants will be recruited from among those who have consented to be contacted for follow-up research. They will be identified in consultation with the field investigators and coordinators of the quantitative study through whom contact will be established.

Efforts will also be made to ensure diversity within the sample, including variation across socioeconomic status, caste, parity, and employment type. This strategy ensures that the qualitative sample captures a wide range of lived experiences while remaining grounded in the cohort population.

### Inclusion criteria

1. Participants eligible for this study will include women who have been recruited in the HiP-India cohort and are either pregnant or within six months postpartum at the time of data collection.
2. They must be residents of one of the three selected heat-prone communities and belong to socioeconomically disadvantaged groups, such as marginalised castes, tribal populations, or low-income households.
3. They should also be regularly exposed to high ambient temperatures due to their daily routines, which may include manual labour, domestic responsibilities, or outdoor work.
4. They should be 18 years or older and willing to provide informed consent.

### Exclusion criteria

1. Women who do not meet these criteria, such as those who were not recruited in the HiP-India cohort, are not pregnant or lactating, have physical or mental health conditions that preclude safe participation, will be excluded from the study.

### Ethics

Ethical approval to conduct the study was given by the:

• Ethical approval was obtained from the Ethics and Research Committee, The George Institute for Global Health, India (Project No. 31/2023)
• Approval was granted by the Institutional Ethics Committee for Translational Health Science and Technology, Institute, Faridabad (THS 1.8.1/(171)
• Approval was granted by the Institutional Ethics Committee, Gurugram Civil Hospital, Haryana (Approval No. GCH/EC/2021/1826/8.12.2023/28.1)
• Approval was granted by the Institutional Ethics Committee for Chhattisgarh Institute of Medical Sciences, Bilaspur (327/C.I.M.S/I.E.C/2024)
• Approval was also obtained from the PIMS Institute Ethics Committee, Department of Community Medicine, Pondicherry Institute of Medical Sciences, Puducherry (IEC Ref No. RC/2024/08), approved on 22.12.2025 for the period 22.12.2025 to 21.06.2028

### Consent

Written informed consent will be obtained from all participants before their involvement in the study. Participants will first be provided with detailed information about the study’s objectives, procedures, potential risks, and expected benefits, both verbally and through the Participant Information Sheet and Consent Form (PISCF) translated into the local language (see Annexures I, II, VI – IX). For non-literate participants, a literate and impartial witness chosen by the participant will be present during the consent process. The qualitative researcher conducting the interview will explain all details of the study in front of this witness. The witness will sign to confirm that the explanation was accurate, and the participant will give consent using a thumb impression. The witness does not give consent on the participant’s behalf. Permission to conduct research will be obtained from the respective hospital authorities and local community representatives.

All interviews and FGDs will be semi-structured and transcribed in English. The digital recordings will be password-protected and stored on secure institutional computers, while hard copies (if any) will be kept in locked cabinets accessible only to the research team. Transcripts will be anonymised before analysis by assigning each participant a unique identification code. The key linking participant identities to these codes will be stored separately and accessible only to the research team.

Electronic data will be encrypted and handled in password-protected programs with restricted access. Access to data will be limited to investigators directly involved in the study. All results will be presented in aggregate form, ensuring that individual participants cannot be identified in any reports or publications. Data containing personal identifiers, including audio recordings, will be securely destroyed after study completion and as per institutional data retention policies.

### Data collection

**Figure 2.**
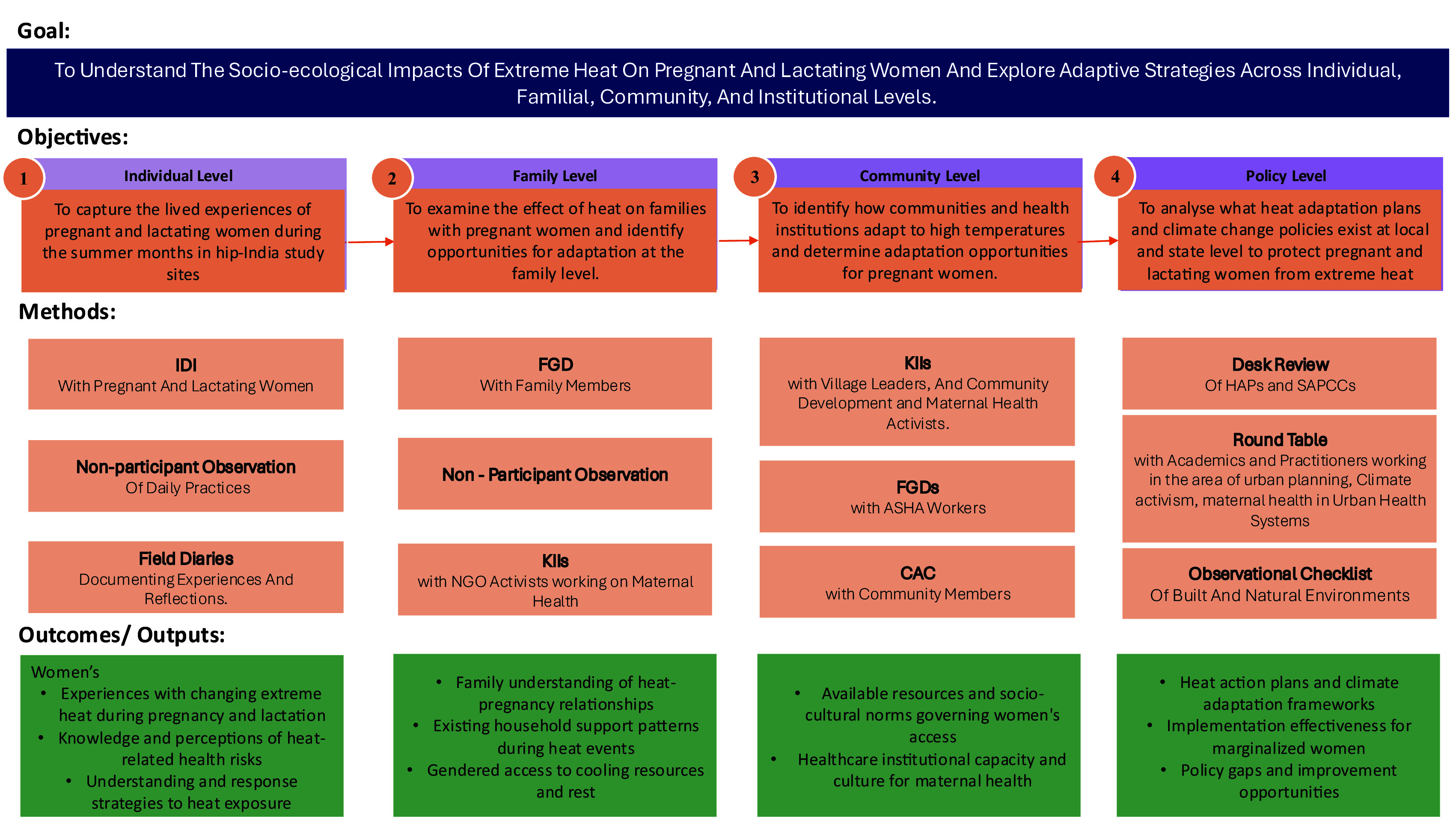
Study Methods.

The socio-ecological framework also guides the study’s methodology by structuring data collection across multiple interconnected levels, enabling a comprehensive analysis of how pregnant and lactating women’s heat adaptation strategies are shaped by and emerge from these layered contextual factors in India ( Table 1, Figure 2).

**Table 1.** Data Collection Methodology.

Socio-Ecological Level	Objective	Participant Group	Number of Participants Per Site	Method of Data Collection
**Individual**	To document the lived experiences of pregnant and lactating women during the summer months at HiP-India study sites, including their understanding of heat-related risks and the strategies they employ to protect themselves.	Currently Pregnant Women	10–15 Women	In-Depth Interviews (IDI)
		Lactating Women	3–5 Women	IDIs
**Family**	To examine the effect of heat on families with pregnant women and identify opportunities for adaptation at the family level.	Family Members of Pregnant/Lactating Women	8–10 Family Members	1–2 Focus Group Discussions (FGD)
**Community**	To identify how actions of community members and workers in health service institutions at the neighbourhood level determine adaptation opportunities for pregnant women.	Key Informants	2–3	Key Informant Interviews (KII)
		Health workers in the public health service system providing services at the level of the neighbourhood-Accredited Social Health Activists (ASHAs), Auxiliary Nurse Midwives (ANMs), Anganwadi Workers (AWWs)	8–10 Health workers	1–2 FGDs
**Policy**	To analyse heat adaptation plans and climate change policies existing at city/district and state levels to protect pregnant and lactating women from extreme heat.	Local stakeholders, such as academics, policy makers, activists, NGO representatives, and institutional actors working in urban planning, climate adaptation, and maternal health sectors	8–12 Members	1 Round Table

**Objective 1: To capture the lived experiences of pregnant and lactating women during the summer months in the study sites of HiP-India
**

i. **IDI:** At the individual level, the study will focus on interviewing 10–15 pregnant and 3–5 lactating women per site to explore their perception of extreme heat and its associated health risks. The interviews will document their lived experiences of heat exposure, its impact on their pregnancy and lactation, and the specific actions they take to protect themselves or treat heat-related illnesses. These include strategies such as modifications in clothing, diet, hydration practices, and rest schedules.
ii. **Field Diaries:** We will maintain these throughout the data collection period to provide descriptive, analytical, and reflective documentation of women’s heat adaptation practices. All researchers will use diary templates to ensure continuous documentation throughout the data collection period, thereby reducing recall bias, which enhances the depth and credibility of the analysis. This will also provide contextual insights that will inform the interpretation of individual adaptations. This approach ensures that individual-level adaptations are interpreted within the backdrop of their own socio-cultural and environmental settings.
iii. **Non-participant observation** of daily practices (see Annexures III) will document women’s heat-related behaviours, activity patterns, and individual adaptation strategies within their natural environments.

**Objective 2: To understand the effect of heat on families with pregnant women and identify opportunities for adaptation at the family level**

i. **FGDs with Family Members:** We will conduct 1–2 focus group discussions per site with 6–8 family members (including spouses) in each site. The discussions will remain relevant by exploring family decision-making processes, resource allocation patterns, and support mechanisms for heat protection. In addition, the study will assess the influence of interpersonal relationships on the ability of women to access cooling resources, prioritise rest, or seek healthcare for heat-related illnesses. However, we recognise that female members may not feel comfortable speaking openly in the presence of male or older family members, which could limit the depth of insight; therefore, we will consider smaller or flexible group formats, including women-only groups, to ensure a safe space for discussion.
ii. **KIIs**: Informant interviews will be conducted with 2–3 village leaders and maternal health activists per site, as well as 2–3 NGO activists working on maternal health per site. Key informants will be purposively selected based on their knowledge of local heat adaptation practices, involvement in maternal health programmes, and understanding of community-level responses to extreme weather events. Discussion topics will include identifying vulnerable populations, existing cooling infrastructure and resources, community awareness and preparedness levels, institutional responses during heatwaves, barriers to accessing heat protection measures, and opportunities for strengthening community-level adaptations.

**Objective 3: To identify how communities and health institutions adapt to high temperatures and opportunities for adaptation for pregnant women**

i. **Community Advisory Committee (CAC):** To ensure meaningful community engagement and cultural relevance, this study will be guided by a CAC comprising 4–6 members per site representing local maternal health workers, women’s advocacy groups and community leaders from heat-prone regions. The CAC will be involved throughout the research process, including reviewing study materials (such as consent forms and interview guides), advising on recruitment strategies, and ensuring that data collection tools are contextually appropriate and ethically sound. Input from the CAC will also inform the framing of research questions and the interpretation of findings, particularly about gender dynamics and adaptive strategies for extreme heat.
ii. **KIIs and FGDs:** This observational data will be complemented by data from 2–3 KIIs at each site with opinion leaders, sarpanches and community-level care providers. The understanding of heat and awareness-building actions and curative interventions of health workers and public and private health care providers at the community level will be examined through 1–2 FGDs per site with Accredited Social Health Activists (ASHA), Anganwadi Workers (AWW) and Auxiliary Nurse and Midwives (ANM) to glean institutional responses to extreme heat, especially concerning pregnant and lactating women.
iii. **Non-Participant Observation:** Data at the community level will be collected using non-participant observation, to develop an understanding of the relationship between exposures to extreme heat and enablers and barriers in the economic, sociocultural, physical and built environments to address their impacts, in communities that the selected sample of pregnant women belongs to.

**Objective 4: To understand how climate change policies on extreme heat influence pregnant and lactating women**

i. **Desk Review**: Using content analysis, we will systematically review publicly available Heat Action Plans (HAPs) and State Action Plans on Climate Change (SAPCCs) across the three study sites. Strategies referencing pregnant women, lactating women, and women with young children, either directly or indirectly (as part of wider vulnerable groups), will be extracted from each document and categorised using Chersich’s climate adaptation framework. ^29^ This review will further examine existing policy frameworks, implementation guidelines, and identify gaps related to maternal health protection during extreme heat events. This analysis will inform our understanding of policy-level factors influencing heat adaptation opportunities for pregnant and lactating women in the study sites.
ii. **Round Tables:** One 3–4-hour roundtable discussion will be convened per site, bringing together 8–12 key local stakeholders, including academics, policy makers, activists, NGO representatives, and institutional actors working in urban planning, climate adaptation, and maternal health sectors. Participants will be purposively selected based on their expertise in climate change impacts and policy, maternal health programming, or community development initiatives relevant to heat adaptation at the local level. The roundtables will facilitate multi-stakeholder dialogue to examine policy implementation challenges, assess gaps between policy frameworks and ground-level realities, and identify opportunities for improving heat adaptation strategies for pregnant women. The roundtables will generate collective insights on systemic barriers and enablers, informing evidence-based recommendations for policy enhancement and implementation improvement.

### Data collection methods

A range of qualitative tools (see Annexures IV,V) will be employed to capture the complexity of women’s lived experiences of extreme heat during pregnancy and lactation. These include semi-structured IDI guides, FGD guides, and a non-participant observation checklist. Each tool is aligned with the study’s socio-ecological framework and designed to generate data on individual, household, and community-level experiences.

All tools will be developed in English and translated into the relevant local languages by trained bilingual researchers. The translations will be reviewed for conceptual and cultural equivalence through back-translation and pretesting. Pilot interviews will be conducted before full deployment, and feedback from CACs will be used to refine language, flow, and contextual relevance.

Data will be collected face-to-face by trained field investigators familiar with the study settings. The IDI guides will be tailored for pregnant and lactating women, focusing on their personal experiences, coping behaviours, and adaptive responses to extreme heat at home and work. While interview guides are designed to focus on heat exposure, participants may raise other climate-related or environmental stressors that are salient to their experiences. In such cases, researchers will allow discussions to evolve organically, documenting these narratives where relevant. These insights will be analysed to understand the broader context of environmental vulnerability, while maintaining analytical focus on heat as the primary exposure of interest. Additional key informant interviews will be conducted with local health providers, traditional healers, and community leaders to capture broader community perceptions of heat-related risks, social norms, and institutional responses.

FGDs will explore collective norms and practices related to pregnancy and lactation during periods of extreme heat, uncovering shared constraints and community-level adaptation strategies. Non-participant observations will systematically document environmental and infrastructural factors such as access to shade, water, green cover, housing materials, cooling devices, and local health services at both household and community levels.

In line with institutional ethical approvals, participants will not receive monetary compensation for participation. However, several measures will be implemented to minimise participant burden, particularly given the involvement of pregnant and lactating women. Interviews and discussions will be scheduled at times and locations convenient to participants, with flexibility to accommodate their daily routines, caregiving responsibilities, and health needs. Where feasible, data collection will take place within participants’ homes or nearby community spaces to reduce travel requirements. Interviews will be kept to a reasonable duration, with provisions for breaks or rescheduling in case of fatigue or discomfort. When data collection is conducted outside their homes, participants will also be provided with refreshments, including drinking water. The research team will ensure that participation does not interfere with essential rest or healthcare, and participants will be reminded of their right to pause or withdraw at any time without consequence.

Data collection will take place in two phases: a primary phase during the summer periods of each site (typically March – July) to capture direct experiences of heat exposure, and a follow-up phase during the non-summer period (typically November – January) to explore reflections on seasonal changes and adaptation sustainability. Data collection will continue until thematic saturation is reached for each site.

### Data analysis and reporting

All audio recordings will be transcribed in the language of data collection and then translated into English. To ensure accuracy and prevent information loss, transcripts will be checked, to ensure the translated text represents the original content of the IDI/FGDs. All transcripts will be de-identified before analysis to protect participant confidentiality. All transcripts will be read and re-read by the research team to build a deep understanding of the data. A comprehensive code list, including codes and sub-codes, will be developed using both deductive (based on the study tools and theoretical framework) and inductive (emerging from the data) approaches. Drawing on insights from field debriefings and researcher reflections, themes will then be identified inductively from the final code list.

The analysis will be iterative and collaborative, involving regular discussions among field researchers and research fellows to refine codes, resolve discrepancies, and ensure consistency across sites. Particular attention will be paid to capturing diverse perspectives, especially those of underrepresented or marginalised participants. Thematic analysis will be conducted using NVivo software by field researchers and research fellows. Data will be analysed using a systematic qualitative approach, specifically thematic analysis guided by both deductive and inductive coding. Observational insights from field supervisors, such as field notes and weekly debrief memos, will be integrated to ensure contextual nuances, non-verbal cues, and environmental factors are captured. This triangulated approach will enhance the richness of the analysis and allow for more nuanced interpretation of the data.

To ensure rigour and trustworthiness, 10–15% of transcripts will be double-coded by 2 independent researchers, and discrepancies will be resolved through consensus. If the consensus is not achieved between independent researchers then final decision will be taken by the principal investigator. Triangulation will be achieved by integrating observational insights from field supervisors, such as field notes and weekly debrief memos, to capture contextual nuances, non-verbal cues, and environmental factors.

The traditional health knowledge (THK) shared by participants during data collection will be referenced against documented texts of the formal systems of traditional medicine like Ayurveda, Unani and Siddha, where possible. Such codified knowledge of traditional systems of medicine is recognized by the Traditional Knowledge Digital Library (TKDL), a government initiative to “protect traditional Indian medicinal knowledge and prevent its misappropriation by international patent offices. ^30^ However, given that oral THK and cultural expressions (CE) including folklore and practices are not adequately protected by Indian intellectual property law, peer reviewed publications and other written and oral outputs from the qualitative study will carry an acknowledgement that the THK and CEs documented in the study are part of the repository of oral knowledge existing in the public domain. ^31^^,^^32^ This knowledge is held, nurtured, and utilised by local communities to which the participants belong and is integral to their economic, cultural, and social fabric.

As part of the broader HiP-India programme, qualitative findings will be integrated with quantitative and clinical data to provide a more comprehensive understanding of heat-related maternal health risks. Specifically, qualitative insights will be used to contextualise and interpret observed associations, identify behavioural and social mechanisms shaping quantitative outcomes, and inform the development of contextually appropriate and policy-relevant adaptation strategies.

### Research team and reflexivity

Interviews and FGDs will be conducted by trained field researchers with backgrounds in public health, sociology, or anthropology, who are fluent in local languages and familiar with the sociocultural context of each site. Training will encompass mock interviews, ethical conduct, and reflexivity exercises, with particular attention to managing sensitive discussions around pregnancy, heat stress, and living conditions.

Reflexivity will be sustained throughout fieldwork through written field journal entries and regular team debriefs, where researchers document assumptions, emotional responses, and potential biases. Structured reflection on positionality—including gender, professional identity, and social and economic distance from the participants—will ensure that data interpretation remains grounded in participants’ own perspectives rather than researcher inference.

Cultural sensitivity is built into the study design at multiple levels. Local researchers will lead data collection, supported by CACs at each site to guide recruitment, culturally appropriate engagement, and contextualised interpretation of findings. Traditional heat adaptation practices will be documented respectfully and in context, ensuring that community knowledge is neither decontextualised nor misrepresented.

Gender and social dynamics will shape how data collection is conducted throughout. Wherever possible, female staff will lead/support interviews with pregnant and lactating women, and formats will be adapted to prioritise participant comfort, privacy, and openness.

### Reporting guidelines

The reporting of findings will follow the COREQ (Consolidated Criteria for Reporting Qualitative Research) framework to promote transparency, rigour, and completeness in qualitative reporting. Key COREQ domains such as researcher reflexivity, study context, participant selection, and data collection and analysis processes will guide both the conduct and presentation of results.

Themes will be reported with illustrative quotations to retain participant voices and linked back to the study objectives and conceptual framework. Findings will be disaggregated by gender, geography, and other relevant demographic factors to reflect intra-group differences.

### Data management and validation

To ensure the accuracy, security, and integrity of the qualitative data collected, all interviews and FGDs will be digitally recorded using encrypted portable devices. These audio files will be transcribed and translated into English by the qualitative research team.

Quality assurance of the transcriptions and translations will be conducted by the researchers and the senior research fellow, who will review the transcripts for accuracy and completeness. To enhance the authenticity and validity of the participants’ narratives, efforts will be made during data collection to clarify meaning and intent in real time.

Member checking will serve as a core validation strategy. Participants and CAC members may be invited to review key thematic insights from their interviews, enabling them to verify the accuracy of representation, clarify potential misinterpretations, and contribute further reflections if necessary.

### Participant risk and ethical considerations

The study involves participation by socioeconomically disadvantaged women, including pregnant and lactating women, who are particularly vulnerable to heat-related health risks. Conducting research during peak summer conditions may increase the likelihood of dehydration, heat stress, or exacerbation of existing health conditions. These risks necessitate a high degree of sensitivity and responsiveness from the research team. Data collection will therefore be scheduled during cooler parts of the day, and participants will be provided with access to hydration as needed. Efforts will be made to ensure interviews are brief, respectful, and located in comfortable settings. Participants will also be clearly informed of their right to withdraw from the interview or study at any time, without providing a reason. Withdrawal from the qualitative study will not affect their participation or data in the quantitative study. If a participant chooses to withdraw from the qualitative component, all recorded data and transcripts pertaining to them will be deleted. The same procedures will apply if a participant withdraws from the entire study. Withdrawal will not affect the care or services they receive from the hospital or any associated health facility. This assurance will be communicated verbally and reinforced during the consent process to uphold participants’ autonomy and well-being.

Researchers will be trained to recognise signs of distress and will pause or reschedule interviews if health concerns arise. If a participant expresses emotional, mental distress and asks for help, the researcher will guide them to appropriate support, including referral to healthcare professionals at the nearest PHC or secondary-level hospital.

### Study risks and mitigation strategies

Several operational risks may arise throughout this study, potentially affecting its implementation and outcomes. Measures to minimise participant burden are embedded within the data collection procedures, including flexible scheduling, location convenience, and participant comfort provisions. Staffing-related risks, such as the departure or unavailability of trained personnel, may disrupt data collection and analysis. To mitigate this, the team will incorporate cross-training and knowledge-sharing strategies to maintain continuity. Fieldwork disruptions caused by overlapping extreme weather events such as floods or cyclones, also pose a risk, particularly in heat-prone regions. Monitoring weather conditions and developing site-specific contingency plans will help ensure that the study can adapt to these challenges.

Given that data collection is scheduled during the summer months, extreme heat conditions present additional risks to both field researchers and participants. To mitigate these, precautionary measures will be implemented, including access to drinking water, ORS, glucose, and traditional cooling drinks. Field researchers will be equipped with protective gear such as caps, scarves, and cotton gloves. All researchers will receive training in basic first aid and will carry first aid kits to address heat-related stress or emergencies.

Data collection will occur at times and locations that prioritise comfort and safety—preferably during the evening or in shaded, well-ventilated spaces. In cases where researchers accompany participants to their workplaces, they may assist with transportation to reduce physical exertion. If a participant experiences mental or physical distress during the study, the researcher will refer them to the nearest secondary-level hospital or local primary healthcare centre (PHC), particularly to antenatal care services for pregnant women.

### Limitations

As a qualitative investigation confined to three selected heat-prone communities, the findings are context-specific and not intended to be representative of all women’s experiences in these sites or outside these sites. The purposive sampling strategy, while suitable for capturing the depth and diversity of lived experiences, may inadvertently exclude subgroups whose perspectives differ from those represented. Logistical disruptions caused by extreme weather events or unforeseen public health challenges may affect the consistency and completeness of data collection across sites. Furthermore, although the research team will undergo training in reflexivity and cultural competence, the positionality of researchers—particularly about gender, caste, and socioeconomic status—may shape participant interactions and influence both data generation and interpretation. These limitations will be carefully considered during analysis and reporting to ensure transparency and contextual integrity.

### Dissemination and communication plan

The study will adopt a multi-tiered dissemination strategy to ensure that findings are accessible, actionable, and relevant to diverse audiences. At the community level, findings will be shared through Village Health and Nutrition Day sessions, participatory workshops, and community-specific reports in local languages, emphasising cost-effective, culturally grounded adaptation strategies. Field researchers’ reflections will be documented through blogs to provide insights into the practical challenges of conducting research in extreme heat, offering valuable lessons for future studies.

Institutional dissemination will include three site-specific reports published on the George Institute’s website, summarising round table discussions with stakeholders and fostering transparency and collaboration. Academic dissemination will involve submissions to peer-reviewed journals focusing on maternal health, climate adaptation, and gender-sensitive policy, with topics including community-based adaptation strategies, socio-ecological drivers of vulnerability, and methodological challenges of fieldwork in high-heat contexts.

To reach broader audiences, findings will also be presented at national and international conferences, webinars, and policy forums. Supplementary materials such as infographics, policy briefs, and visual summaries will be developed to support evidence uptake amongst the public, policymakers and practitioners.

## Conclusions/Discussion

As extreme heat events intensify under climate change, pregnant and lactating women in LMICs face increasingly urgent health risks from heightened physiological demands, limited cooling access, and entrenched socioeconomic disadvantages. ^9^^,^^11^^–^^13^^,^^15^^,^^27^ Despite these compounded vulnerabilities, the intersection of extreme heat and maternal health remains critically underexplored in global and national adaptation frameworks. ^21^^,^^22^^,^^24^ This study addresses this critical gap by generating grounded, qualitative evidence on how pregnant and lactating women in heat-prone Indian communities experience, navigate, and adapt to high ambient temperatures.

Adopting a socio-ecological approach, the research moves beyond individualised risk models to examine how social roles, household dynamics, built environments and development policies shape adaptive capacity. ^14^ The study’s participatory design, including community dissemination, stakeholder engagement, and researcher reflections, strengthens relevance and legitimacy while documenting underutilised community practices often overlooked in formal HAPs. By capturing these contextual processes, the study provides insights into how vulnerability is produced, how coping strategies are shaped, and where interventions can meaningfully support women.

Climate adaptation must move beyond technical fixes to address the determinants that shape vulnerability for this critically underserved population. Thus, this research is timely and needed to illuminate pathways toward equitable adaptation, informing gender-responsive, culturally grounded strategies that engage meaningfully with the lived realities of those most affected by extreme heat.

## Data Availability

No data associated with this article. The supplementary materials for this study are available via Zenodo: Heat in Pregnancy (HiP-India): Qualitative study protocol and supplementary materials. https://doi.org/10.5281/zenodo.18218269. ^33^ This repository contains the following underlying materials:
• Interview and focus group discussion guides used for qualitative data collection. • Observation checklists and field diary templates used to document contextual and environmental factors. All materials are available under the Creative Commons Attribution 4.0 International (CC BY 4.0) licence.
