# Supplementary material for: To gain insights into heat adaptations among women during the antenatal period in India -The Heat in Pregnancy Project (HiP-I) India- Qualitative Study Protocol
Source: Wellcome Open Res. 2026 Apr 24;11:93. Originally published 2026 Feb 5. [Version 2] doi: 10.12688/wellcomeopenres.25507.2 (PMC13125996; doi:10.12688/wellcomeopenres.25507.2)
Supplement: Peer review history

# Reviewer report: Reviewer response for version 2

**DOI:** 10.21956/wellcomeopenres.29158.r154053

**Published:** 2026-04-28

**Authors:** Supriya Mathew

**Affiliations:** 1 Menzies School of Health Research, Charles Darwin University, Alice Springs, Australia

The authors have addressed my comments.

Is the study design appropriate for the research question?

Yes

Is the rationale for, and objectives of, the study clearly described?

Yes

Are sufficient details of the methods provided to allow replication by others?

Partly

Are the datasets clearly presented in a useable and accessible format?

Not applicable

Reviewer Expertise:

NA

I confirm that I have read this submission and believe that I have an appropriate level of expertise to confirm that it is of an acceptable scientific standard.

# Reviewer report: Reviewer response for version 1

**DOI:** 10.21956/wellcomeopenres.28097.r147991

**Published:** 2026-03-13

**Authors:** Supriya Mathew

**Affiliations:** 1 Menzies School of Health Research, Charles Darwin University, Alice Springs, Australia

The protocol outlines a well-designed qualitative study addressing a gap in evidence on how heat affects pregnancy and maternal health. It is part of a larger research program, that could benefit from in depth on the ground qualitative data. The study will cover interviews with pregnant and lactating women about their lived experiences of heat, focus group discussions with family caregivers, key informant interviews with health workers and local officials, and non-participant observations across three locations. By integrating perspectives from community members, practitioners, policymakers, and researchers, the study complements existing epidemiological research and provides a more comprehensive understanding of heat-related maternal health risks.

 The proposal may need attention on the below points

1. The plain language summary states that the study will take place during the summers of 2025–2026. As the protocol is being submitted/reviewed in 2026, this phrasing reads somewhat confusingly and may imply that part of the study has already occurred. Consider rewording this to indicate that the study will take place over two consecutive summer seasons. It could be that 2025 data collected has progressed given ethics approvals have been obtained. In that case, it might be useful to mention that and even state that learnings from the 2025 recruitment have informed the current protocol.
2. Three study sites have been selected, which provides opportunity to compare and contrast and share findings across the sites. It would be helpful for the authors to provide additional contextual information about these locations (e.g., climate zones, population size, or key socio-environmental characteristics). This would assist readers in understanding the differences between sites and whether the data are intended to be analysed collectively across sites or separately by site to account for contextual variation.
3. The statement that the global literature on heat and health has focused on high-income contexts, with limited attention to the lived experiences of women in LMICs, is valid. However, the references cited here appear to be reviews of quantitative studies. I would like to draw attention to another recent systematic review funded by Wellcome that identified very limited qualitative research globally on heat, maternal health, and pregnancy experiences, highlighting an important gap in the literature (Grimshaw et al., 2026). Grimshaw F, Thakur R, Gebremedhin A, Matthews Quandamooka V, Farrant B, Shepherd C, Phillips C, Wyrwoll CS, Mathew S. *Hot weather, maternal health, and pregnancy experiences, impacts and responses: A systematic review of global qualitative research.* Women Birth. 2026;39(1):102152. (Ref 1)
4. The protocol mentions purposive sampling of 20–25 participants for the lived experience component of the study. However, it is not entirely clear how these participants will be recruited. The protocol states that participants will be recruited through PHC centres, but it also refers to a quantitative study in which participants have already provided consent. It would be helpful for the authors to clarify whether participants will be recruited directly through PHC centres or from the existing quantitative study cohort. If the latter, please clarify whether participants previously provided consent to be re-contacted for follow-up or related studies.
5. It would be helpful for the authors to clarify whether participants will be remunerated or compensated for their time. In addition, given that the study involves pregnant and lactating women, please indicate whether any  support measures, such as childcare arrangements or flexible interview scheduling, will be provided where needed. Further clarification on how the research team will ensure that participation and data collection do not impose additional burden on pregnant or lactating mothers would also strengthen the protocol.
6. While sharing people’s lived experiences, is there a chance cultural knowledge may be shared? Are there IP principles that need to be factored? Also, do cultural backgrounds restrict women from having conversations with non-local researchers or male researchers? It would be helpful to mention these if measures have been considered. 
7. The protocol notes that logistical disruptions from extreme weather events or unforeseen public health challenges may affect data collection. In practice, participants may also wish to discuss recent weather-related hazards that are more salient to them than heat exposure. It would be helpful for the authors to clarify how the research team will manage situations where participants prioritise discussing other hazards. For example, will the interviews remain strictly focused on heat, or could a broader multi-hazard perspective be incorporated if such issues arise during discussions?
8. Given this qualitative study is part of a larger project how will the qualitative information be integrated with the overall findings

Is the study design appropriate for the research question?

Yes

Is the rationale for, and objectives of, the study clearly described?

Yes

Are sufficient details of the methods provided to allow replication by others?

Partly

Are the datasets clearly presented in a useable and accessible format?

Not applicable

Reviewer Expertise:

Environmental health, mixed methods research, climate change, rural and remote research, health systems research

I confirm that I have read this submission and believe that I have an appropriate level of expertise to confirm that it is of an acceptable scientific standard, however I have significant reservations, as outlined above.

## References

[1] : Hot weather, maternal health, and pregnancy experiences, impacts and responses: A systematic review of global qualitative research. *Women and Birth* .2026;39(1) : 10.1016/j.wombi.2025.102152 10.1016/j.wombi.2025.102152 41401689

# Response

**Published:** 2026-04-15

**Authors:** Karthika Kumar

**Affiliations:** Not Applicable, India

The protocol outlines a well-designed qualitative study addressing a gap in evidence on how heat affects pregnancy and maternal health. It is part of a larger research program, that could benefit from in depth on the ground qualitative data. The study will cover interviews with pregnant and lactating women about their lived experiences of heat, focus group discussions with family caregivers, key informant interviews with health workers and local officials, and non-participant observations across three locations. By integrating perspectives from community members, practitioners, policymakers, and researchers, the study complements existing epidemiological research and provides a more comprehensive understanding of heat-related maternal health risks.

**Response:** *We sincerely thank the reviewer for their careful reading of our manuscript and for their constructive and insightful feedback. We are pleased that the reviewer finds the study well-designed and valuable in addressing an important evidence gap. We have revised the manuscript accordingly and respond to each point below.*

 The proposal may need attention on the below points

 The plain language summary states that the study will take place during the summers of 2025–2026. As the protocol is being submitted/reviewed in 2026, this phrasing reads somewhat confusingly and may imply that part of the study has already occurred. Consider rewording this to indicate that the study will take place over two consecutive summer seasons. It could be that 2025 data collected has progressed given ethics approvals have been obtained. In that case, it might be useful to mention that and even state that learnings from the 2025 recruitment have informed the current protocol.

**Response:** *We appreciate this observation. We have revised the plain language summary to clarify that the study is being conducted over two consecutive summer seasons, rather than implying retrospective data collection. Where relevant, we now also clarify that ethical approvals were obtained prior to initiation and that early field insights have informed refinements to the protocol.*

 Three study sites have been selected, which provides opportunity to compare and contrast and share findings across the sites. It would be helpful for the authors to provide additional contextual information about these locations (e.g., climate zones, population size, or key socio-environmental characteristics). This would assist readers in understanding the differences between sites and whether the data are intended to be analysed collectively across sites or separately by site to account for contextual variation.

**Response:** *Thank you for this suggestion. We have expanded the description of the three study sites to include key contextual characteristics, such as climate profiles, urban/peri-urban typologies, and socio-environmental vulnerabilities (Page 10; lines 105 -111). We have also clarified that analysis will be conducted both within and across sites, allowing for contextual comparison while preserving site-specific nuance.*

 The statement that the global literature on heat and health has focused on high-income contexts, with limited attention to the lived experiences of women in LMICs, is valid. However, the references cited here appear to be reviews of quantitative studies. I would like to draw attention to another recent systematic review funded by Wellcome that identified very limited qualitative research globally on heat, maternal health, and pregnancy experiences, highlighting an important gap in the literature (Grimshaw et al., 2026). Grimshaw F, Thakur R, Gebremedhin A, Matthews Quandamooka V, Farrant B, Shepherd C, Phillips C, Wyrwoll CS, Mathew S. Hot weather, maternal health, and pregnancy experiences, impacts and responses: A systematic review of global qualitative research. Women Birth. 2026;39(1):102152. (Ref 1)

**Response:** *We are grateful for this recommendation and have now incorporated the cited systematic review (Grimshaw et al., 2026) into the introduction (page 9; lines 59 -65). This strengthens our argument by explicitly highlighting the global scarcity of qualitative research on heat and maternal health, further reinforcing the relevance of our study.*

 The protocol mentions purposive sampling of 20–25 participants for the lived experience component of the study. However, it is not entirely clear how these participants will be recruited. The protocol states that participants will be recruited through PHC centres, but it also refers to a quantitative study in which participants have already provided consent. It would be helpful for the authors to clarify whether participants will be recruited directly through PHC centres or from the existing quantitative study cohort. If the latter, please clarify whether participants previously provided consent to be re-contacted for follow-up or related studies.

**Response:** *We agree that this required clearer articulation. The manuscript now explicitly states that participants will be primarily recruited from the HiP-India cohort, who have previously consented to participation and re-contact (Page 10; Lines 114-117). We also clarify the role of PHCs and frontline health workers in facilitating recruitment, particularly for participants with limited facility access.*

 It would be helpful for the authors to clarify whether participants will be remunerated or compensated for their time. In addition, given that the study involves pregnant and lactating women, please indicate whether any  support measures, such as childcare arrangements or flexible interview scheduling, will be provided where needed. Further clarification on how the research team will ensure that participation and data collection do not impose additional burden on pregnant or lactating mothers would also strengthen the protocol.

**Response:** *Thank you for raising this important ethical consideration. We have added clarification that (Page 18; lines 390-408):*

- *Participants will not receive compensation/reimbursement in line with institutional ethical guidelines.*
- *Data collection will be designed to minimise burden through flexible scheduling, shorter interview durations, and where feasible, accommodations such as childcare considerations or home-based interviews.*
- *Additional safeguards (e.g., conducting interviews during cooler hours, ensuring hydration, and prioritising participant comfort) have been further emphasised.*

**  **While sharing people’s lived experiences, is there a chance cultural knowledge may be shared? Are there IP principles that need to be factored? Also, do cultural backgrounds restrict women from having conversations with non-local researchers or male researchers? It would be helpful to mention these if measures have been considered.

**Response:** *We appreciate this important point. We have strengthened the manuscript to reflect (Page 17; Lines 370-387):*

- *Sensitivity to cultural knowledge and practices, including careful handling and contextual representation of traditional adaptation strategies.*
- *The role of local researchers and Community Advisory Committees (CACs) in ensuring culturally appropriate engagement.*
- *Consideration of gender dynamics, including matching interviewer characteristics (e.g., female field researchers/staff where appropriate) and adapting methods to ensure participant comfort and openness.*

The protocol notes that logistical disruptions from extreme weather events or unforeseen public health challenges may affect data collection. In practice, participants may also wish to discuss recent weather-related hazards that are more salient to them than heat exposure. It would be helpful for the authors to clarify how the research team will manage situations where participants prioritise discussing other hazards. For example, will the interviews remain strictly focused on heat, or could a broader multi-hazard perspective be incorporated if such issues arise during discussions?

**Response:** *Thank you for highlighting this practical consideration. We have clarified that while interviews are guided by a focus on heat, the study will remain responsive to participant priorities (Page 16 ; Lines 305 -310) . If other climate-related hazards emerge as salient, these will be documented and analysed as part of a broader understanding of environmental stressors and adaptive capacity, while maintaining analytical focus on heat.*  

 Given this qualitative study is part of a larger project how will the qualitative information be integrated with the overall findings

**Response:** *We have now elaborated on how qualitative findings will be integrated within the broader HiP-India programme (Page 17; Lines 382 – 387). Specifically, qualitative insights will:*  

- *Provide contextual interpretation of quantitative findings*
- *Help explain mechanisms and behavioural pathways linking heat exposure and health outcomes*
- *Inform policy-relevant recommendations grounded in lived experience*

 
*This integration strengthens the mixed-methods contribution of the overall project.*

# Reviewer report: Reviewer response for version 1

**DOI:** 10.21956/wellcomeopenres.28097.r147994

**Published:** 2026-03-06

**Authors:** Paulo H. N. Saldiva

**Affiliations:** 1 Department of Pathology, University of São Paulo, São Paulo, Brazil

**Your Report**

 This proposal explores at the intersection of climate change, reproductive health, and gendered vulnerability. The epidemiological literature now provides robust evidence that gestational heat exposure is associated with increased risks of preterm birth, low birth weight, and stillbirth. While the physiological mechanisms underlying these associations—dehydration, hemoconcentration, endothelial stress, impaired uteroplacental perfusion, and inflammatory activation—are increasingly well characterized, information on how pregnant and lactating women in low-resource settings experience, interpret, and respond to heat stress in their daily lives are not hitherto fully clarified

 The proposed qualitative study does not attempt to re-establish epidemiological associations or identify the pathophysiolgy of heat-associated low birth weight;  rather, it addresses the implementation gap between biological vulnerability and adaptive practice. The geographic scenatio is South Asia—where climate projections indicate escalating heat intensity and duration—pregnancy occurs within environments shaped by gendered labor roles, constrained autonomy, limited cooling infrastructure, and variable access to prenatal care. These structural conditions may plausibly modify the response to thermal limits.

 The study’s socio-ecological framework is appropriate, recognizing that thermoregulatory strain in pregnancy is not solely a biological phenomenon but one modulated by housing conditions, occupational exposure, caregiving responsibilities, and institutional responsiveness. By conducting interviews during and shortly after the heat season, the investigators strengthen ecological validity and minimize recall distortion. The inclusion of caregivers, health workers, and local officials adds triangulation and enhances interpretive depth.

 The proposal does have limitations. The sample size is relatively small, and recruitment primarily through primary health centers may introduce selection bias toward women already engaged with the health system. This may underrepresent the most marginalized individuals. However, for qualitative inquiry aimed at understanding lived experience, adaptive strategies, and structural barriers, depth and contextual richness are more important than statistical representativeness. The investigators demonstrate awareness of these constraints and transparently delimit the study population where necessary.

 The anticipated outputs—documentation of coping strategies, identification of structural barriers, and characterization of culturally embedded practices—are highly relevant to policy translation.  Given the accelerating frequency of heatwaves in South Asia and the documented reproductive risks associated with heat exposure, advancing from observational epidemiology toward context-sensitive adaptation research is both scientifically justified and ethically imperative. Despite its modest scale, this study has the potential to generate actionable knowledge that can inform culturally grounded, gender-responsive interventions and strengthen inclusive climate adaptation frameworks.

 In view of its relevance, conceptual clarity, methodological appropriateness for its aims, and strong alignment with current scientific and policy needs, the proposal merits approval.

Is the study design appropriate for the research question?

Yes

Is the rationale for, and objectives of, the study clearly described?

Yes

Are sufficient details of the methods provided to allow replication by others?

Yes

Are the datasets clearly presented in a useable and accessible format?

Not applicable

Reviewer Expertise:

Human Pathology, Experimental Pathology and Environmental Pathology. High Impact infectious diseases. Air Pollution health effects. Climate and Health

I confirm that I have read this submission and believe that I have an appropriate level of expertise to confirm that it is of an acceptable scientific standard.
